# Proceedings of the Suffolk District Medical Society

**Published:** 1879-04

**Authors:** T. M. Rotch

**Affiliations:** Secretary


### PROCEEDINGS OF THE SUFFOLK DISTRICT MEDICAL SOCIETY.

T. M. ROTCH, M.D., SECRETARY.

OCTOBER 26, 1878. Sixty-three members were present, the President, Dr. C. D. Homans, in the chair.

*Retroversion of the Uterus complicated with Pregnancy.*—Dr. J. R. Chadwick read a series of cases of Retroversion of the Uterus complicated with Pregnancy. Reserved for publication.

*Œsophagotomy.*—Dr. G. W. Gay read a case of œsophagotomy, which was published in the *Journal* for March.

*Amputation of the Hip-Joint.*—Dr. Gay also reported the following case: The patient was a stout, fleshy man, forty-two years of age. He first noticed a small, oval bunch just above the internal condyle of the left femur three or four years ago. At first the growth was gradual, but of late it had increased very rapidly, being a third larger at the time of the operation than it was three months previously. He entered the City Hospital October 11, 1878, under the care of Dr. Thorndike. The left thigh measured thirty-eight inches in circumference. The tumor was hard, lobulated, and extended from the knee to the great trochanter. The pain was so severe that life was a burden to the man, and he was willing to take any risk for the sake of relief. October 18th, Dr. Thorndike amputated the leg at the hip-joint by a large, outside skin flap extending from the middle of Poupart's ligament to the tuberosity of the ischium, and an inner muscular flap. The femoral artery was tied previous to making the flaps; vessels were seized as soon as divided, so that the hæmorrhage was not excessive. Not over half a dozen ligatures were used. It should be stated that the sphincters were dilated, and the hand passed into the rectum previous to the operation, with a view to controlling the hæmorrhage by pressure upon the common iliac artery. The vessel was easily reached, but owing to the large amount of fat and soft tissues about the artery, and to the fact that the arm of the assistant would have been in the operator's way, that plan was abandoned, and the femoral artery tied about three inches below its commencement. The patient did well for two days, when pyæmic symptoms set in, and he died on the fourth day. The amputated limb weighed fifty-four pounds.—Dr. E. G. Cutler reported that a gross examination of the thigh showed it to be made up of fat tissue, which was elastic, more or less gelatinous, and was arranged distinctly in alveoli surrounded by a fibrous tissue bearing blood-vessels. The disease began in the thigh, posteriorly, at the back of the intercondyloid space, and encroached upon the tissues in a general upward course in all directions; the bone was but slightly involved behind and below. In no place had the growth broken through the hard portion of the bone into the cancellated structure. A microscopical examination showed the tumor to be composed of a basement hyaline transparent substance, in which, imbedded in little grooves, were cells of various shapes, round, oval, stellate, and caudate. These cells had but a single wall. In other places the basement membrane was fibrous, and the cells were close together. The cells were differently arranged in different parts,—in some places close together, in others more separated. The tumor was an enchondroma.

*Imperfect Plumbing.*—Dr. H. I. Bowditch presented a specimen of lead pipe which had been taken from a house where a case of typhoid fever had occurred, and later one of diphteria. A leak was detected by the peppermint test at the junction of this pipe with the soil-pipe, it being connected with the latter only by means of putty and wire.

*Oleic Acid.*—Dr. E. W. Cushing called the attention of the society to the advisability of applying, in certain cases where the hypodermic injection of morphia is objected to, a solution of morphia in oleic acid, rubbed over the painful part. He stated that he had found it as efficacious as hypodermic injections, but that it did not act quite so quickly.—Dr. Ingalls remarked that he had tried this remedy in cases of painful joints, some years ago, without any apparent effect, but that he had probably made use of a poor preparation.—Dr. Cushing replied that very marked results had been obtained from the application of a mixture of morphia and mercury, and that he knew of a case where quite severe symptoms of poisoning had arisen after the application of thirty minims of a solution of morphia in oleic acid of the strength of Magendie's solution.

*Felons.*—Dr. Bradford stated that he had noticed in his treatment of felons that, after the incision, the wound healed more rapidly when dressed with carbolized oil than by the old method of simple poulticing; he is now in the habit of first washing the wound in a solution of carbolic acid, and then applying cotton-wool soaked in carbolized oil, the whole being enveloped in sufficient wool to retain the flow of pus; the dressing is not removed for twenty-four hours, and is then reapplied.

NOVEMBER 30, 1878. *Electrolysis of Uterine Fibroid.*—Dr. E. Cutter reported the case of a woman, forty-four years of age, with a uterine fibroid, through which he passed a current of electricity by introducing one of the needles into the tumor by the rectum, and the other through the abdominal wall. At the time of the operation the pulse rose to 130 beats per minute; after the operation considerable pain was reported, and the temperature at one time rose to 102° F. Relief from pain and from hæmorrhage was claimed for the operation.

*Excision of the Hip-Joint,*—Dr. E. H. Bradford reported a series of cases of excision of the hip-joint, and exhibited the specimens.

*Optico-Ciliary Neurotomy.*—Dr. Wadsworth exhibited a patient on whom he had divided the optic and ciliary nerves of the left eye to avert the danger of sympathetic ophthalmia. The man was a boot-black, forty years of age. Fourteen years ago cataract had developed in both eyes. The cataract was removed from the left eye thirteen years ago, but some two years later its sight gradually failed again, as he was told, from separation of the retina. Ten years ago the cataract was extracted from the right eye. Both eyes were quiet until the 10th of September last; then the left became red, he had photophobia and lachrymation, and soon there came on tenderness over the upper part of the ciliary body, though without much pain. He was treated as an out-patient at the Infirmary, and toward the end of October was apparently well, and resumed his work. He had been at work but a week, however, when the redness of the left eye and photophobia returned, and three or four days later, November 7 h, he went to Dr. Wadsworth, who found in the left eye moderate circumcorneal congestion, with tenderness on pressure in the upper ciliary region; its pupil was large, but no reflex could be obtained from the fundus; it was completely blind. The right eye was sensitive to light, but appeared otherwise in good condition. Atropine, rest, and avoidance of light did not relieve the symptoms, and there also began to be an appearance of sparks before the right eye. November 12th, under ether, an incision was made over the internal rectus of the left eye, a strabismus hook passed beneath the muscle, and two sutures carried from within outward through muscle and conjunctiva a little back from the insertion of the former. The tendon was then divided ½ ''' from its insertion, and the cut extended freely upward and downward through the conjunctiva and Tenon's capsule. The attached end of the tendon was seized by forceps, and the globe rolled outward, while curved scissors were pushed between globe and capsule, and the optic nerve divided as in enucleation. Very little bleeding followed, but as a measure of precaution the lids were closed, and pressure made with a sponge for two or three minutes. The curved scissors were again introduced to divide the remaining ciliary nerves and arteries, and then the globe could be readily rolled into a position which brought the stump of the opticus almost directly in front, and made it evident that all the ciliary nerves had been severed. The eye was replaced, the internal rectus united to its tendon by the sutures, and a compressive bandage applied. There was very little exophthalmos at this time. The evening after the operation there was pain in the head, but not particularly about the eye, and apparently attributable to the ether. The next day the exophthalmos was considerable; it seemed to be owing to swelling of the tissues behind the eye, not to hæmorrhage. The lids were ecchymosed, but not swollen; the conjunctiva was dark red, somewhat swollen and ecchymotio. The cornea was clear. Aside from a little tenderness over the conjunctival wound, there was no pain, nor was there any subsequently. During the next few days the exophthalmos gradually subsided, but chemosis of the lower part of the conjunctiva came on. The sutures were removed on the fourth, the bandage was omitted on the ninth day. The chemosis diminished for several days; then, probably from slipping of the bandage, and consequent uneven pressure, increased again somewhat. During the past week, under iced compresses, it had disappeared. Meanwhile the eye had been constantly gaining in freedom of motion, until, but for a little insufficiency on the side of the divided muscle, it moved as far and as readily as the other. The cornea was tested on the second day, and found completely anæsthetic, and it had remained so; its transparency had been unimpaired from the first. On the twelfth day a small clot of blood was seen at the bottom of the anterior chamber. Nothing was discovered to account for its presence, and two days later it had been absorbed. There still remained some conjunctival congestion, but in other respects the eye appeared as well as before the operation. Dr. Wadsworth said that the operation was a comparatively new one. Schoeler, of Berlin, had published an account of ten cases in which he had so operated during the last part of 1877 and first part of 1878. Hirschberg and Schweigger had also performed the operation successfully. It was remarkable that after division of all the nerves supplying the interior of the eye and the cornea, and division of the greater part of the arteries going to the interior of the eye, it should still preserve its vitality. Ulceration of the cornea Schoeler had indeed observed in two of his cases, but it was not extensive, was painless, and healed readily under appropriate treatment; it could, moreover, in both cases, be fairly referred to a defect in the operation or after-treatment. It could not yet be stated with certainty that the ciliary nerves might not reunite, as so frequently happened in the case of section of other nerves, and thus opportunity for the excitation of sympathetic ophthalmia be again presented. But the anatomical conditions were here such as to make reunion, at the least, very improbable. The ciliary nerves were small, ran through loose tissues, and could readily contract, and even though a few of them might be held in position by connections with Tènon's capsule, yet the almost constant movement of the eye within the capsule must of itself make reunion very unlikely. Remembering that after enucleation it was sometimes impossible to wear an artificial eye with comfort; that it was often difficult to procure an eye which matched the other well; that the cost was considerable for a poor person; that the movement of an artificial eye was in the most favorable case much limited, while often there was no movement, it seemed that optico-ciliary neurotomy, as Schoeler had named the new operation, was likely to take the place of enucleation to a very considerable extent.

*Santa Barbara.*—Dr. E. L. Parks made some interesting remarks in regard to the climate of Santa Barbara, and its value as a place of resort for consumptives, which will be published.—*Boston Med. and Surg. Journal.*

